# Why Even More Clinical Research Studies May Be False: Effect of Asymmetrical Handling of Clinically Unexpected Values

**DOI:** 10.1371/journal.pone.0065323

**Published:** 2013-06-25

**Authors:** Matthew James Shun-Shin, Darrel P. Francis

**Affiliations:** International Centre for Circulatory Health, National Heart and Lung Institute, Imperial College, London, United Kingdom; University of Westminster, United Kingdom

## Abstract

**Background:**

In medical practice, clinically unexpected measurements might be quite properly handled by the remeasurement, removal, or reclassification of patients. If these habits are not prevented during clinical research, how much of each is needed to sway an entire study?

**Methods and Results:**

Believing there is a difference between groups, a well-intentioned clinician researcher addresses unexpected values. We tested how much removal, remeasurement, or reclassification of patients would be needed in most cases to turn an otherwise-neutral study positive. Remeasurement of 19 patients out of 200 per group was required to make most studies positive. Removal was more powerful: just 9 out of 200 was enough. Reclassification was most powerful, with 5 out of 200 enough. The larger the study, the smaller the proportion of patients needing to be manipulated to make the study positive: the percentages needed to be remeasured, removed, or reclassified fell from 45%, 20%, and 10% respectively for a 20 patient-per-group study, to 4%, 2%, and 1% for an 800 patient-per-group study. Dot-plots, but not bar-charts, make the perhaps-inadvertent manipulations visible. Detection is possible using statistical methods such as the Tadpole test.

**Conclusions:**

Behaviours necessary for clinical practice are destructive to clinical research. Even small amounts of selective remeasurement, removal, or reclassification can produce false positive results. Size matters: larger studies are proportionately more vulnerable. If observational studies permit selective unblinded enrolment, malleable classification, or selective remeasurement, then results are not credible. Clinical research is very vulnerable to “remeasurement, removal, and reclassification”, the 3 evil R's.

## Introduction

We already know that most published research findings are false, as elegantly explained by Ioannidis [1]. Clinical medical research may be worse. Medical training produces standardised knowledge, values, beliefs, and behaviours. Clinical readers might like to confirm this by checking how they would answer the questions below:

### Scenario 1

You see a student nurse about to document into the medical records an oxygen saturation by pulse oximetry of 85%. The patient is ambulant, looking pink and feeling well. All previous values have been > = 97%. Do you:

- Immediately confine to bed and initiate 100% oxygen.
- Document 85% and request tests for possible pulmonary embolism,
- Remeasure the oxygen saturation yourself, and document the new value instead?

### Scenario 2

In the middle of a busy clinical day you are called to an unfamiliar ward and asked to teach some students about pneumothorax. On the ward there is a collection of four anonymised teaching radiographs in the pneumothorax folder. On three you can see the pneumothorax; the fourth looks normal to you even on close inspection. Do you:

- Show all four radiographs and claim you can see pneumothoraces in all four,
- Show all four and admit you cannot see the pneumothorax on the fourth,
- Show the three with visible pneumothoraces?

### Scenario 3

A General Practitioner refers a patient with an asymptomatic ejection systolic murmur to your cardiology department for echocardiography. It shows echocardiographically very severe aortic stenosis and a ventricle beginning to fail. What do you do?

- Wait for the patient to leave and then send a report to the GP saying asymptomatic aortic stenosis.
- Call the cardiac surgeons to refer for valve surgery saying severe aortic stenosis
- Ask the patient in more detail about symptoms, asking them what they mean by no symptoms, and what activities they can do, to look for unmentioned symptoms.

Clinical practice runs smoothly and consistently because most doctors would take the third option in each case. These behaviours of remeasurement, removal, and reclassification of patients are the occult oil in the medical machine.

But how much harm could they do in clinical research? Imagine three researchers in separate laboratories around the world who believe that patients in two groups differ in their values of a variable. Each researcher proclaims high clinical and research standards. Dr A is particularly fastidious, taking care to remeasure any initial measurements where they are inconsistent with the clinical picture. Dr B is especially scrupulous about bias in research and tries to prevent even a few patients who have other intercurrent diseases from distorting results. Dr C realises that unaided clinical judgement may be poor at classifying patients and that test results may be better guidance.

In this study we examine how much of an effect their meticulous, well-intentioned clinical habits would have on the results of a study of the difference between those two patient groups.

## Methods

### Simulating the effect of remeasurement, removal, and reclassification

We studied the effect of three doctors' clinical biases by running large numbers of simulations. The underlying data were normally distributed values drawn from a single consistent population, i.e. no difference between groups. We simulated a series of study sizes from 20 patients per group to 1000 per group. For each group size, each doctor's type of inadvertent manipulation, and each degree of manipulation, we ran 100,000 simulations.

**Figure 1:**
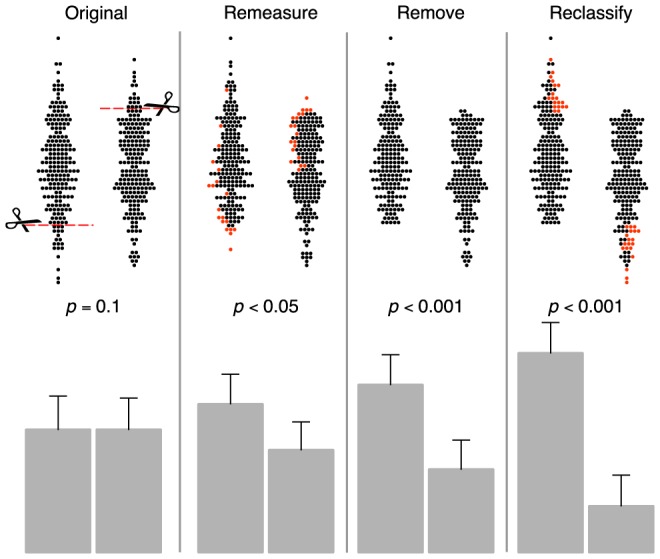
A clinical researcher, who believes that two groups differ may, at the time of acquiring a clinically surprising value in an individual patient may elect to remeasure, remove, or reclassify the patient based on the rationalisations cited above.

We simulated each manipulation in turn, applying it to the values considered clinically surprisingly low in one group and surprisingly high in the other (Figure 1, upper panels).

For remeasurement, those selected patients underwent repeat measurement with the variable simulated to have an intraclass correlation coefficient of 0.5. The new measurement was used instead of the original if it was less extreme. For removal, the selected patients were simply removed. Dr B would explain that these individuals almost certainly had some other condition which made them unrepresentative of their groups. For reclassification, the patients were transferred between groups. Dr C would explain that they must have been wrongly classified on superficial clinical criteria and their correct classification was obvious once the quantitative data were available.

We should emphasise that we are not assessing a formal, statistically valid, and neutral process for handling of outlier [1], but merely the consequences of applying the clinical common sense that is carried out by every doctor every day.

### Determining how much manipulation is needed

We observed how many patients had to be manipulated before the majority of simulations showed Student's t-test to be statistically significant. The pooled variance version and a criterion of p<0.05 was used. Because the manipulation of values from one end of the distribution will lead to a skewed distribution we also repeated the simulation using the non-parametric Mann-Whitney U-test.

### Tadpole Test, a statistical test to detect these manipulations

**Figure 2:**
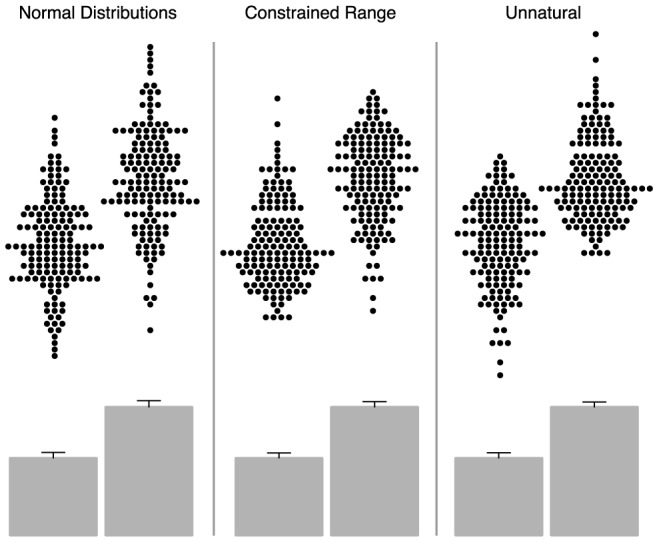
In many medical situations the distributions of patients in each group is similar. For example, height in men and women has a similar shape (left panel). In other situations there may be a constrained range with the groups clustered at opposite ends of the spectrum, commonly showing a long tail from each group extending towards the other (middle panel). A third pattern does not easily arise in nature. In this, the tails in each group point away from each other (right panel), leaving the heads meeting, like kissing tadpoles.

In many medical situations the distributions of patients in each group is similar. For example, height in men and women has a similar shape (Figure 2, upper left panel). In other situations there may be a constrained range with the groups clustered at opposite ends of the spectrum, commonly showing a long tail from each group extending towards the other. For example, hair colour in Norway may be predominantly blond, but with a spectrum extending all the way to dark; meanwhile in Italy it is predominantly dark but again with a wide spectrum extending all the way to blond (Figure 2, upper middle panel).

A third pattern does not easily arise in nature. In this, the tails in each group point away from each other (Figure 2, upper right panel), leaving the heads meeting, like kissing tadpoles.

This visual pattern arises in our 3 manipulations (Figure 1) because they selectively attenuate one tail in one group, and the opposite tail in the other.

Numerically, this skew is increased in the group with the larger mean, and decreased in the group with the smaller mean; implausible for most clinical variables. A straightforward “Tadpole test” for this phenomenon is to check whether the skews of the two groups are surprisingly different in magnitude and direction. It can be done by calculating the D'Agostino z-score for skew in each group [2]. If that z-score for the higher-mean group is more than 1.64√2 above the z-score for the other group, the Tadpole test is significant at the p<0.05 level (Equation 1). Our simulations ran the Tadpole test alongside the Student's t-test.

### 



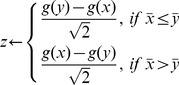
(1)
*Where g() refers to the D'Agostino transformation of the skew,* 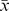 *and* 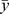 *are the means of x and y respectively.*

### Software

Simulations and graphing were performed using the free and open-source “R” Statistical Environment version 2.15.0 and graphing package ggplot2 version 0.9.0. We have made our simulation software free and open-source permitting cost-free replication of results by any interested reader using Windows, Mac OS X, or Linux.

Dot plots [3] were generated using R, but we additionally provide spreadsheets to approximate this using Microsoft Excel and the free and open-source LibreOffice.

## Results

### Effects of remeasurement, removal, and reclassification

Dr A's remeasurement redistributes some patients with initially surprising values closer to their clinically expected region. The visual effect is subtle. There is no distinct cut-off since a few manipulated patients – whose remeasurement gave more-extreme results – retain their original values. Dr B's manipulation by removal is more visually dramatic, amputating the lower end of one group and the upper end of the other. Dr C's manipulation of reclassification is similarly dramatic and, additionally, thickens the tails of the kissing tadpoles. The histograms in the lower panel illustrate the increasing differences, and statistical significance of the difference, between the resulting group means.

### How much manipulation is needed?

**Figure 3:**
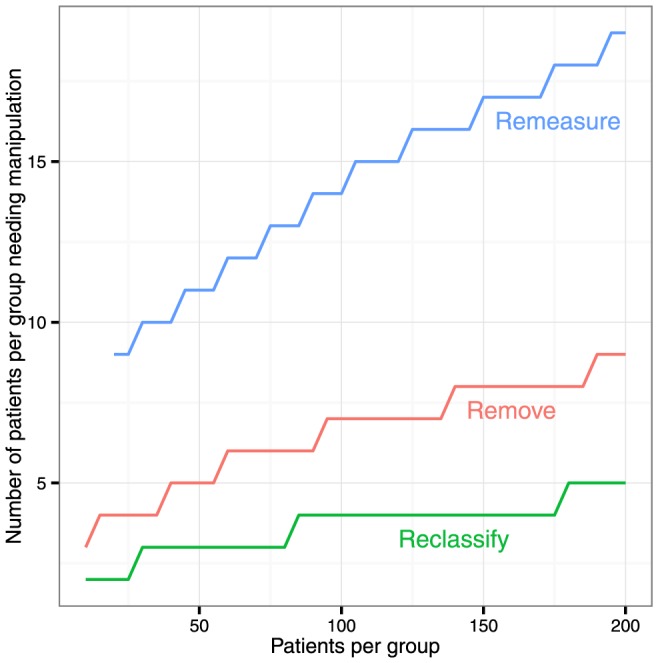
The number of patients per group needing to be remeasured, removed, or reclassified to, on average, make an otherwise neutral study positive by the Student t-test.

The number of patients per group needed to be manipulated to make most study results positive is shown in Figure 3.

Remeasurement in a 20 patient-per-group study required 9 patients per group to be remeasured before most of the 100,000 simulated studies became positive. Removal was more powerful, requiring only 4 patients per group. Reclassification is even more powerful requiring on average only 2 patients per group.

For larger studies, more patients need to be manipulated in order to make the results positive most of the time. At 200 patients per group, the numbers per group needing manipulation were 19, 9, and 5 respectively.

**Figure 4:**
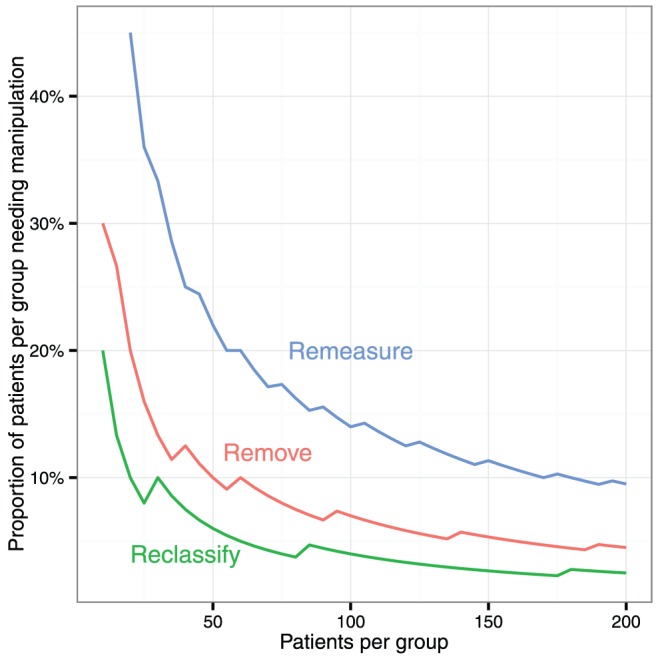
The proportion of patients per group needing to be remeasured, removed, or reclassified to, on average, make an otherwise neutral study positive by the Student t-test.

However, the *proportion* of patients needing manipulation fell progressively as study size grew (Figure 4). At 20 patients per group, manipulations were needed in 45%, 20% and 10% of patients respectively. By 800 it was just 4%, 2% and 1%.

**Figure 5:**
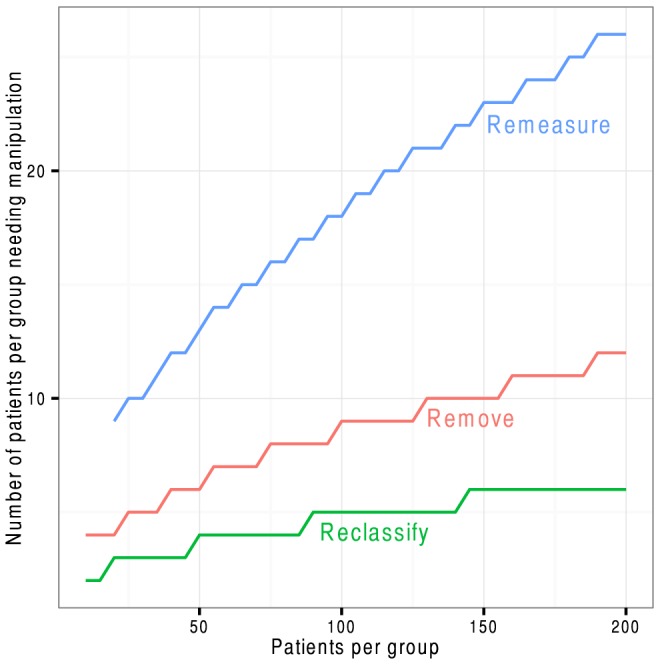
The number of patients per group needing to be remeasured, removed, or reclassified to, on average, make an otherwise neutral study positive by the non-parametric Mann-Whitney U-test.

**Figure 6:**
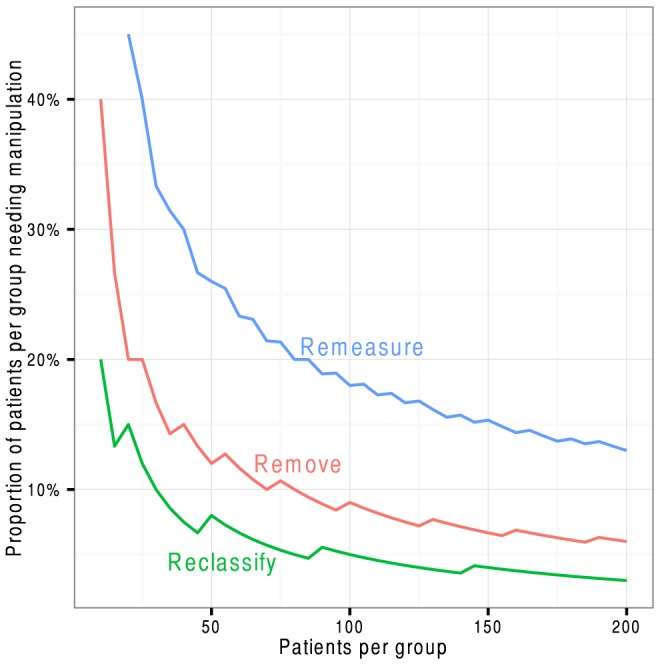
The proportion of patients per group needing to be remeasured, removed, or reclassified to, on average, make an otherwise neutral study positive by the non-parametric Mann-Whitney U-test.

Most clinicians analyse most data with standard parametric tests as shown above. Sometimes a dot-plot is shown and a reviewer may notice the skew of the distribution and request a non-parametric test. Our study found that the Mann-Whitney U-test too was railroaded by these three manipulations. At 200 patients per group, the numbers per group needing remeasurement, removal, and reclassification were 26 (13%), 12 (6%), and 6 (3%) respectively (Figure 5 and 6).

### The Tadpole test for detection of these manipulations

At group sizes above 100, the Tadpole test is able to detect these three manipulations.

The degree of manipulation required before the Tadpole test became positive most of the time is shown in Table 1.

**Table 1:** Number of patients that can be manipulated before, on average, the manipulation is detected by the “tadpole test”.

Manipulation	Patients per group	
	100	200
Remeasure	9 (9%)	8 (4%)
Remove	5 (5%)	3 (1.5%)
Reclassify	3 (3%)	6 (3%)

## Discussion

Even mild application of clinical common sense during the acquisition of data can destroy the validity of an unblinded study. This is because clinicians commonly use the term “outlier” value asymmetrically to the situation of being surprised by a measurement in the full knowledge of the clinical context of that particular patient.

Tiny amounts of any one of three normal clinical behaviours – remeasurement, removal, or reclassification – are enough to falsely generate apparent differences between identical groups. Only a very small minority of data needs adjustment, and nothing need be fabricated. All that is needed is knowledge of which group the patient is in, and a background of medical training that emphasises selecting the measurements that fit the clinical picture.

### Normal clinical practice

In the clinical vignettes in Box 1 most doctors choose the third option. Remeasurement, removal, and reclassification are inbuilt into us because medicine is a craft rather than a science. We handle difficult situations, with incomplete or unreliable information, somehow synthesising confident clinical action with coherent justification so that patients and other staff do not suffer through unnecessary doubt.

It may be difficult for clinicians moving repeatedly between medical practice and research to remember which behaviour is correct at any given time especially if they have never recognised the distinction.

### Why are larger studies *more* sensitive to bias?

Minor matters generally have proportionately minor consequences. However, this study shows that particular handling of a very few clinically unexpected values can have major effects on the result of a study. The three types of cleaning-up are normal clinical behaviours. Even a clinical researcher who condemns two of them might still defend the third, denying that it is harmful.

Although in larger studies the number of patients needing to be manipulated is larger, the proportion of patients needing to be manipulated is actually smaller.

This means that it is the larger studies that are the more threatened by this subtle source manipulation.

Clinicians are drilled to expect larger studies to be more reliable. This is formalised in guidelines which remind readers incorrectly [4] that large non-randomised studies have the same status as randomised controlled trials.

Whilst at first this may seem counter-intuitive, it is in fact to be expected. Statistical tests assess if the data differs from the null hypothesis by chance alone, but are unable to determine if this arises from a true change or from bias. Larger studies are able to detect smaller effects.

The greater detection capacity of larger studies is agnostic to whether this effect is a true effect or just bias. Larger study sizes do not change the balance between genuine differences and bias, as explanations for positive findings.

The visual effect of these manipulations can be striking but most studies do not show individual data points, nor make available the raw numerical values. From the bar graphs (Figures 1 and 2, lower panels), the reader would have no inkling that the groups differed only in the handling of a few outliers.

We set a high bar for the manipulation to be considered as affecting the results – that at least 50% were considered positive at the nominal p<0.05 threshold. Lower rates would still significantly distort the scientific literature [5], and would require even less manipulation. For example, we found that only 2 or 3 patients per group need be removed in a 100 or 200 patient-per-group study to make 10% of studies be considered positive.

### The Tadpole test

The pattern of tails of two groups pointing away from each other should be particularly eye-catching because in nature if there is a tendency of the two tails to be different in direction they should be pointing towards each other, for the reasons described above.

The Tadpole test is an automatic statistical tool that can be applied with any raw data to highlight suspicious cases. Since it returns a z-score, it can easily be aggregated across multiple studies to perceive manipulations that are individually subtle. For systematic manipulation the Tadpole test is so sensitive that it picks up some cases before the manipulation has even made the study positive.

Interested readers can use the supplemental online spreadsheets on their own data to test for this very human weakness.

### Implications

Dot-plots, or raw data-points, are the gold-standard currency of quantitative research. They reveal everything that could be gleaned from a bar chart, and much more, including the distribution of the data and, to motivated readers, the numerical values [6]. Authors should show these unless they have something to hide. Simple, free tools for their display using free and open-source spreadsheets such as Libreoffice [7], the free and open-source “R” statistical environment [8], and Microsoft Excel, are attached in the online supplements (Supplementary File 1 & 2).

Observational clinical research is more vulnerable than widely supposed to small biases of types routine and necessary for healthy clinical practice. This may partly explain why observational studies may overstate differences between groups even when there is no publication bias [9]–[11]. Clinical researchers need particular guidance not to spread clinical practice into research environments. Reporting of studies should honestly cover possible sources of bias and steps taken to reduce it [12].

### Limitations

In this study we used simulation. This was so that we could be confident of the nature of the underlying distribution.

Real-life clinical data is not always normally distributed. In these circumstances authors generally transform data so that the distribution is approximately normal. Our study addresses data after such transformation.

Some observational studies may consider more than two groups, and therefore be analysed by ANOVA, the multi-group equivalent of the t-test. Because remeasurement, removal, and reclassification increase between-group variance and decrease within-group variance, ANOVA is vulnerable too.

This study does not address the actual prevalence of bias in the observational study literature because this is already known to by high [10], [13], [14] and detailed case examples are available [15]. Instead it only shows that minor activation of three behaviours routinely required in clinical practice is enough to cause false findings.

### Conclusions

Clinical research is conducted by physicians who rely on remeasurement, removal, and reclassification in daily practice. Results can very easily be biased enough to generate positive results falsely. Inadvertent bias affecting a tiny fraction of patients has overwhelming effects. Large studies generate false positive findings at an even smaller proportion of manipulation that do small ones. Authors should be encouraged to show raw data in dot-plots rather than conceal them in bar-charts. Raw data permits some manipulations to be detected by eye or through statistical testing. Clinical researchers should beware the three evil R's – remeasurement, removal, and reclassification.

### Highlights

#### What is already known on this topic

Bias is the scourge of clinical research, and many observational studies and methodologically inadequate randomised trials have succumbed.

In their daily practice clinicians appropriately remeasure, remove, and reclassify inconsistent or outlier data-points.

#### What this study adds

Clinical research is highly susceptible to such practices, and if even a small remnant remains otherwise neutral studies will appear statistically significant

Strict assurance of blinding is required for results to be credible.

## Supporting Information

File S1 **An Excel Spreadsheet compatible with both Microsoft Excel and Libreoffice Calc for generating dot-plots for up to 6 groups with 300 points per group.**
(XLS)

File S2 **An Excel spreadsheet compatible with both Microsoft Excel and Libreoffice Calc for calculating the “Tadpole Test” for two groups of data, and generating a dot-plot.**
(XLS)
